# Association of Selected Anthropometric Indices With Diabetic Kidney Disease Among Iranian Adults With Diabetes

**DOI:** 10.1002/edm2.70282

**Published:** 2026-07-12

**Authors:** Mehrsadat Miri, Maryam Karimi Ghahfarokhi, Shahrzad Mohseni, Ali Golestani, Samaneh Akbarpour, Mahnaz Pejman Sani, Ozra Tabatabaei‐Malazy

**Affiliations:** ^1^ Non‐Communicable Diseases Research Center, Endocrinology and Metabolism Population Sciences Institute Tehran University of Medical Sciences Tehran Iran; ^2^ School of Medicine Tehran University of Medical Sciences Tehran Iran; ^3^ Department of Biostatistics, School of Allied Medical Science Shahid Beheshti University of Medical Sciences Tehran Iran; ^4^ Endocrinology and Metabolism Research Center, Endocrinology and Metabolism Clinical Sciences Institute Tehran University of Medical Sciences Tehran Iran; ^5^ Department of Endocrinology, Shariati Hospital Tehran University of Medical Sciences Tehran Iran

**Keywords:** diabetic kidney disease, Iran, lipid accumulation product, triglyceride‐glucose‐body mass index, triglyceride‐glucose index, visceral adiposity index, waist‐triglyceride index

## Abstract

**Aims:**

This study evaluated the association and discriminative ability of several lipid‐ and adiposity‐related indices—triglyceride‐glucose (TyG), TyG‐body mass index (TyG‐BMI), waist‐triglyceride index (WTI), visceral adiposity index (VAI) and lipid accumulation product (LAP)—with diabetic kidney disease (DKD) among Iranian adults with diabetes.

**Methods:**

In this nationwide cross‐sectional study, data were obtained from 3272 diabetic individuals aged ≥ 25 years who participated in the 2021 world health organization (WHO) STEPwise approach to Noncommunicable Disease Risk Factor Surveillance (STEPS) survey. Survey‐weighted multivariable logistic regression models were applied for each index, adjusting for demographic, lifestyle, clinical and medication‐related factors. Discriminative performance was evaluated using receiver operating characteristic (ROC) curves.

**Results:**

Overall, 27.9% of participants had DKD. Individuals with DKD exhibited significantly higher mean values across all studied indices (*p* < 0.001). In adjusted analyses, each one‐standard deviation (SD) increase in TyG, TyG‐BMI, WTI, VAI and LAP was associated with 36%, 20%, 27%, 24% and 27% higher odds of DKD, respectively (all *p* < 0.001). All indices demonstrated moderate discriminative performance, with area under the curve (AUC) values ranging from 0.696 to 0.704; however, no significant differences were observed in their discriminatory ability.

**Conclusion:**

In this large sample of Iranian adults with diabetes, low‐cost indices were associated with DKD and may help identify high‐risk individuals, particularly in resource‐limited settings. However, their moderate discriminative performance suggests limited standalone diagnostic utility, and longitudinal studies are needed to establish their predictive value.

## Graphical abstract

Selected anthropometric indices showed independent associations with diabetic kidney disease in Iranian adults with diabetes, but only moderate discriminatory ability, supporting their use as adjunctive markers rather than standalone diagnostic tools.

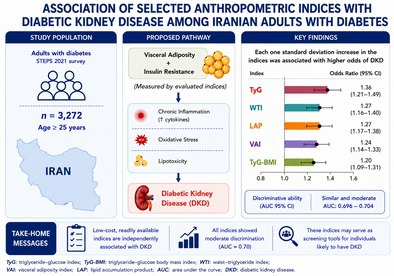

## 1 Introduction

According to the International Diabetes Federation (IDF), diabetes affected about 11% of adults aged 20–79 years in 2024 and is projected to rise substantially by 2050 [1]. Diabetic kidney disease (DKD) is one of the most important microvascular complications of diabetes, affecting approximately 20%–40% of patients [2, 3]. The age‐standardized incidence rate (ASR) of DKD among type 2 diabetic patients in Iran has notably increased from 20.02 per 100,000 in 1990 to 49.03 per 100,000 in 2019 [4]. DKD is a leading cause of end‐stage renal disease (ESRD) and is associated with heightened risks of cardiovascular disease (CVD), diabetic foot complications and increased mortality [5]. Beyond individual health impacts, DKD imposes a substantial economic burden on healthcare systems and significantly impairs patients' quality of life [6]. Because established renal damage is difficult to reverse, timely detection and prevention remain critical [7].

DKD arises from a complex interplay of metabolic disturbances, haemodynamic alterations, chronic inflammation and oxidative stress [8, 9]. Among these, insulin resistance (IR) plays a central role in the development of kidney injury. IR may promote and contribute to glomerulosclerosis, tubulointerstitial inflammation and renal fibrosis [10, 11]. In parallel, abnormal lipid metabolism and visceral adiposity are increasingly recognized as important contributors to renal damage through lipotoxicity, chronic low‐grade inflammation and adverse metabolic signalling [12]. These pathways may accelerate kidney dysfunction in individuals with diabetes and increase the likelihood of DKD. At present, the hyperinsulinemic–euglycemic clamp (HIEC) is regarded as the gold standard for assessing IR. However, this technique requires highly standardized procedures, is costly and invasive, and is therefore not feasible for large‐scale epidemiological studies [13]. The homeostatic model assessment of insulin resistance (HOMA‐IR) is a widely used indirect alternative; however, it requires measurement of insulin concentrations and is not applicable to patients with diabetes who are receiving exogenous insulin [14]. Consequently, there is a pressing need to identify simple, cost‐effective and reliable markers in both clinical practice and population health surveillance [15].

Traditional anthropometric measures such as body mass index (BMI) and waist circumference (WC) provide useful but limited information about adiposity. BMI does not distinguish between fat mass and lean mass, and WC alone cannot fully capture the metabolic activity of visceral fat. Therefore, more refined indices that integrate anthropometric and biochemical information may better reflect the metabolic disturbances relevant to DKD [16]. In recent years, several indices have been proposed as simple and low‐cost markers of insulin resistance and visceral adiposity, including the triglyceride‐glucose (TyG) index, TyG‐body mass index (TyG‐BMI), waist‐triglyceride index (WTI), visceral adiposity index (VAI) and lipid accumulation product (LAP). Since IR [17] and visceral adiposity [18] are closely linked to DKD pathophysiology, these indices may help identify individuals at high renal risk beyond what conventional anthropometric measures can provide.

The TyG index is widely used as a surrogate marker of IR, while TyG‐BMI combines glucose, lipid and general adiposity‐related information [19]. In contrast, WTI, VAI and LAP are more closely related to central obesity, visceral fat dysfunction and lipid accumulation [20]. Evidence indicates that the TyG index and TyG‐BMI are associated with albuminuria, reduced estimated glomerular filtration rate and DKD [21]. The WTI, another marker of central obesity and dyslipidemia, has been less extensively studied [22], and its relationship with DKD remains unclear. The VAI, a well‐established indicator of visceral adiposity, captures key metabolic disturbances—including inflammation, IR, dyslipidemia and β‐cell dysfunction [23], offering significant implications for patient management [24]. Similarly, the LAP reflects central lipid accumulation and metabolic impairment [25]. Notably, elevated VAI and LAP values have been linked to a higher prevalence of chronic kidney disease (CKD) and DKD [26, 27].

Despite this evidence, data on the relationship between these indices and DKD remain limited in Iranian adults. Therefore, this study aimed to evaluate the associations of TyG, TyG‐BMI, WTI, VAI and LAP with DKD, and to assess their discriminative performance in a large adult population participating in the 2021 WHO STEP‐wise approach to Non‐communicable Disease (NCD) Risk Factor Surveillance (STEPS) as well as their discriminative ability in Iranian individuals with diabetes.

## 2 Materials and Methods

### 2.1 Study Design and Participants

This cross‐sectional study utilized data from the STEPS 2021 survey [28], a large‐scale, population‐based study conducted in Iran to assess risk factors of non‐communicable diseases [29]. In summary, the STEPS survey employed a systematic cluster sampling design to recruit adults aged ≥ 18 years from both urban and rural areas across all 31 provinces, ensuring national representativeness. Data collection involved three sequential phases: in phase 1, structured questionnaires were administered to gather self‐reported information on demographic characteristics, dietary habits, medical history, physical activity, quality of life, lifestyle counselling, cancer screening, injuries, tobacco and alcohol consumption and household assets. Written consent was obtained during this phase; phase 2 involved physical measurements, including weight, height, waist and hip circumferences, blood pressure (BP) and heart rate, all performed by trained healthcare professionals using pre‐calibrated instruments; and phase 3 entailed participants' blood and urine samples for biochemical analysis [29].

A total of 28,821 individuals were selected for the survey; however, participation rates varied by study phase: 27,874 completed phase 1, but 27,745 completed phase 2, and 18,119 completed phase 3. In the STEPS survey, participants were excluded if they had psychological conditions impairing questionnaire completion, physical limitations preventing physical or laboratory assessments, or were pregnant [28, 29].

**FIGURE 1:**
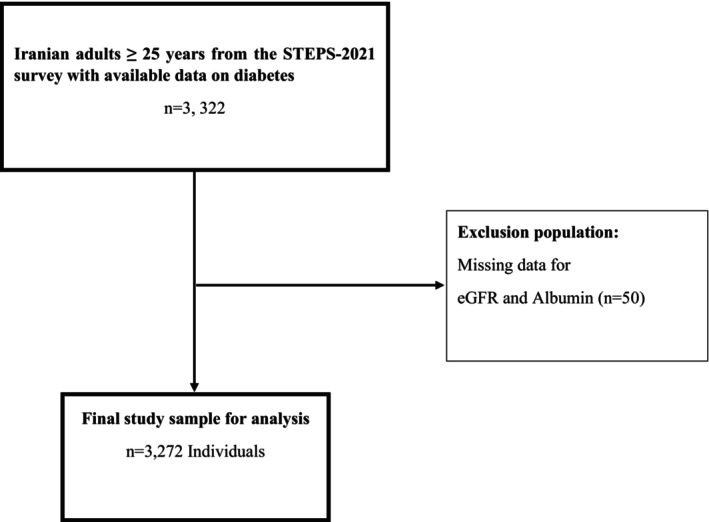
The flowchart of the study population selection.

For the present analyses, only diabetic participants aged 25 years or older with available data on chronic kidney disease, anthropometric and lipid profile across urban and rural areas were included. Of the 3322 participants with diabetes who enrolled in the study, we excluded 50 individuals due to missing data for eGFR and urine albumin. Ultimately, 3272 participants were included for further analysis (Figure 1).

Anthropometric measurements were conducted using a calibrated scale (Innofit, China) with 1 cm height and 1 kg weight accuracy. BMI was calculated as weight divided by height squared (kg/m^2^). Waist circumference was measured at the midpoint between the lower rib margin and iliac crest using a flexible tape (1 cm precision). Following a 15‐min rest period, blood pressure was measured using an automated digital sphygmomanometer (Beurer BM 20, Germany), with three consecutive readings taken at 3‐min intervals; the mean of the last two readings was used for analysis. Biochemical measurements were performed on blood and urine samples collected from participants aged ≥ 25 years who completed Phase 3. Samples were collected, stored at 4°C, and transported in vaccine transfer boxes, ensuring transfer times did not exceed 18 h prior to laboratory analysis. Fasting blood sugar (FBS), glycosylated haemoglobin (HbA1c), and serum level of total cholesterol (TC), high‐density lipoprotein cholesterol (HDL‐C), and TG were measured using an auto‐analyser (Cobas C311 Hitachi). Low‐density lipoprotein cholesterol (LDL‐C) was calculated using the Friedewald formula: LDL‐C = TC−[HDL‐C + triglyceride/5]. Non‐HDL‐C was derived by subtracting HDL‐C from TC. Morning spot urine samples were obtained for sodium, albumin and creatinine measurements. Urinary albumin was assessed using the Prestige Premium 24i analyser [28].

### 2.2 Definition of Variables

Demographic variables included age (categorized as < 40, 40–59 and ≥ 60 years), sex (male/female), residence (urban/rural). Education level was assessed by years of completed schooling and grouped into three categories: < 7 years, 7–11 years and ≥ 12 years. Occupational status was categorized as employed and unemployed. Smoking was defined as any occasional or daily use of tobacco products including cigarettes, electronic cigarettes, pipes, hookah or smokeless tobacco, during a person's lifetime [30]. Physical activity was considered adequate if it exceeded 600 Metabolic Equivalent of Task (MET)‐minutes/week and inadequate if below this threshold, following WHO guidelines [31]. Tanaka's equation was applied to estimate salt intake, previously validated against 24‐h urine collections in a subsample, with estimates adjusted upward by 10% to account for sweat‐related sodium losses [32]. Given that 97.3% of study participants exceeded 5 g/day, high salt intake was defined as daily consumption above the study participants' median of 9.79 g/day.

Diabetes was defined as FBS ≥ 126 mg/dL (7 mmol/L), HbA1c ≥ 6.5% or use of antihyperglycemic medications [33]. Glycemic control was classified by HbA1c levels, with < 7% indicating controlled diabetes and ≥ 7% indicating uncontrolled diabetes. Diabetes duration was categorized as < 10 years or ≥ 10 years. Diabetes treatment was grouped as oral agents only, insulin only, or combination therapy based on participant‐reported use [34]. Dyslipidemia was defined as self‐reported use of lipid‐lowering medications or at least one abnormality in lipid markers as follows: TC ≥ 200 mg/dL (5.2 mmol/L), TG ≥ 150 mg/dL (1.7 mmol/L), LDL‐C ≥ 130 mg/dL (3.4 mmol/L), non‐HDL‐C ≥ 160 mg/dL (4.1 mmol/L), and HDL‐C < 50 mg/dL (1.29 mmol/L) in females and < 40 mg/dL (1.03 mmol/L) in males [35]. Hypertension (HTN) was defined as systolic blood pressure (SBP) ≥ 140 mmHg or diastolic blood pressure (DBP) ≥ 90 mmHg, or self‐reported use of anti‐hypertensive medication [36]. CVD was defined as self‐reported history of myocardial infarction, angina, coronary revascularization (bypass surgery, angioplasty or stenting), or stroke diagnosed by a healthcare professional.

Medication use—including statins, aspirin, angiotensin converting enzyme inhibitors (ACE‐Is), and angiotensin receptor blockers (ARBs)—was considered positive if participants reported current use. Given the predominance of HMG‐CoA reductase inhibitors (statins) among participants, statin use was specifically highlighted in the medication analysis. eGFR was calculated based on CKD‐EPI Creatinine Equation [37]. DKD was defined as estimated glomerular filtration rate (eGFR) < 60 mL/min/1.73 m^2^ or urine albumin‐to‐creatinine ratio (UACR) ≥ 30 mg/g in diabetic individuals [38].

The anthropometric indices were calculated as follows:

- TyG index = Ln [TG (mg/dL) × FPG (mg/dL)/2] [21]
- TyG‐BMI = Ln [TG (mg/dL) × FPG (mg/dL)/2] × BMI (kg/m^2^) [21]
- LAP for male = [(WC (cm) − 65)] × TG (mmol/L) [26]
- LAP for female = [(WC (cm) − 58)] × TG (mmol/L) [26]
- VAI for male = [WC (cm)/(39.68 + 1.88 × BMI (kg/m^2^))] × [(TG (mmol/L)/1.03)] × [1.31/HDL‐C (mmol/L)] [23]
- VAI for female = [WC (cm)/(39.58 + 1.89 × BMI (kg/m^2^))] × [(TG (mmol/L)/0.81)] × [1.52/HDL‐C (mmol/L)] [23]
- WTI = Ln [TG (mg/dL) × WC (cm)/2] [39]

### 2.3 Statistical Analysis

Demographic and clinical variables were summarized using survey‐weighted proportions with 95% confidence intervals (95% CI) for categorical variables and survey‐weighted means with 95% CI for continuous variables, accounting for the complex sampling design and sampling weights of the STEPS survey. The relationships between categorical variables were assessed using the chi‐square test, and the means of continuous variables were compared between independent groups using the independent samples *t*‐test.

Univariable logistic regression analyses were conducted to examine the crude associations between individual risk factors and the presence of DKD. These analyses were performed to describe unadjusted relationships and were not used for variable selection.

To ensure comparability of effect estimates, all continuous index variables (TyG, TyG‐BMI, WTI, VAI and LAP) were standardized (*z*‐scores; mean = 0, SD = 1) prior to inclusion in regression models.

Pairwise Pearson correlation coefficients were calculated to assess the strength and direction of associations among adiposity‐related and insulin resistance‐related indices (TyG, TyG‐BMI, WTI, VAI and LAP), as well as BMI and waist circumference. The correlation matrix was used to evaluate shared variance and potential multicollinearity among variables prior to multivariable modelling.

In the next step, multivariable logistic regression was used to examine associations between candidate risk factors and the presence of DKD. Although a wide range of demographic, clinical, lifestyle, anthropometric, laboratory and medication variables were collected, the selection of covariates for adjustment was primarily guided by prior clinical and epidemiological knowledge and evidence from the literature. Given the relatively large number of candidate variables and the presence of correlations among them, a stepwise logistic regression procedure was additionally applied as an exploratory variable screening tool to support model parsimony and reduce redundancy, rather than as the primary basis for confounder selection or inference. A *p*‐value of 0.20 was used as the criterion for both entries into and removal from the stepwise procedure [40, 41].

Robust standard errors were applied to account for potential heteroscedasticity and clustering in the data.

Given that indices such as TyG‐BMI, WTI, VAI and LAP directly incorporate BMI and/or waist circumference in their calculation formulas, the simultaneous inclusion of these variables would result in conceptual overlap and information redundancy. Therefore, BMI and waist circumference were excluded from subsequent analyses, and the correlation matrix was presented to illustrate the extent of shared information among these measures. Additionally, very strong correlations were observed among WTI, VAI and LAP, indicating that these indices capture closely related aspects of visceral adiposity and metabolic dysfunction despite differences in their computational approaches. All of the indices—TyG, TyG‐BMI, WTI, VAI and LAP—were evaluated separately in individual multivariable models due to high collinearity among them and with other variables included in the model. The final adjusted models included the following covariates: Age (years), Gender, Residency, Employment status, Educational status (years), Ever Smoking, Dietary salt intake (gr/day), DM duration (years), SBP, Aspirin use, Anti‐hypertensive medications, Anti‐diabetic medications.

All statistical analyses were performed using R software (version 4.4.1; release 2023.06.0). Associations were reported as odds ratios (OR) and adjusted odds ratios (AOR) with corresponding 95% CIs. A *p*‐value < 0.05 was considered statistically significant.

To evaluate the discriminative ability of clinical indices for the presence of DKD, receiver operating characteristic (ROC) curves were plotted for each index (TyG, TyG‐BMI, LAP, VAI and WTI). This approach allows assessment of how well each index can distinguish between participants with and without DKD. The area under the curve (AUCs) was reported along with their 95% CI to indicate the precision of the estimates.

Pairwise comparisons of the area under the receiver operating characteristic curves (AUCs) were performed to assess differences in the discriminative performance of the studied indices (TyG, TyG‐BMI, LAP, VAI and WTI). Since all ROC curves were derived from the same study population, DeLong's test for correlated ROC curves was used for statistical comparison. Given the multiple pairwise comparisons performed, adjustment for multiple testing was applied [42] using the Benjamini–Hochberg false discovery rate (FDR) method to control for type I error inflation [43]. Both unadjusted and FDR‐adjusted *p*‐values were considered in the interpretation of results, and a *p*‐value < 0.05 was considered statistically significant.

The present study was reviewed and approved by the Research Ethics Committees of Endocrine and Metabolism Research Institute at Tehran University of Medical Sciences, Tehran, Iran (ID: IR.TUMS.EMRI.REC.1403.092). This study was conducted in accordance with relevant institutional guidelines, regulations and the Declaration of Helsinki.

## 3 Results

### 3.1 Baseline Characteristics of the Study Participants

A total of 3272 participants with diabetes and a mean age of 57.74 (95% CI: 57.28, 59.20) years were included in the present analysis comprising of 2359 individuals (72.09%) without DKD and 913 individuals (27.9%) with DKD. Baseline participant characteristics stratified by the DKD status are presented in Table 1.

**TABLE 1:** Survey‐weighted descriptive characteristics of participants with diabetes mellitus by diabetic kidney disease status.

Variable	Overall estimate	Diabetes mellitus (%, *n* = 3272)		*p*
		No DKD (*n* = 2359)	DKD (*n* = 913)	
Age (years)	57.74 (57.28, 59.20)	55.82 (55.31, 56.33)	62.73 (61.85, 63.60)	< 0.001
Sex				
Male	59.26 (57.36, 61.12)	59.52 (57.29, 61.72)	58.56 (54.95, 62.07)	0.286
Female	40.71 (38.88, 42.64)	40.48 (38.28, 42.71)	41.44 (37.93, 45.05)	
DM duration (years)				
< 10	74.84 (73.12, 76.48)	79.05 (77.13, 80.85)	63.92 (60.35, 67.35)	< 0.001
≥ 10	25.16 (23.52, 26.88)	20.95 (19.15, 22.87)	36.08 (32.65, 39.65)	
Residency				
Urban	74.01 (72.32, 75.64)	74.88 (72.90, 76.75)	71.76 (68.40, 74.90)	0.222
Rural	25.99 (24.36, 27.68)	25.12 (23.25, 27.10)	28.24 (25.10, 31.60)	
Educational status (years)				
< 7	60.74 (58.84, 62.61)	57.06 (54.80, 59.29)	70.34 (66.86, 73.60)	< 0.001
7–11	14.51 (13.19, 15.93)	15.81 (14.20, 17.55)	11.12 (9.05, 13.60)	
≥ 12	24.75 (23.11, 26.47)	27.13 (25.15, 29.22)	18.54 (15.79, 21.65)	
Employment status				
Unemployed	24.32 (22.71, 26)	26.74 (24.79, 28.79)	17.99 (15.36, 20.97)	< 0.001
Employed	75.68 (74, 77.29)	73.26 (71.21, 75.21)	82.01 (79.03, 84.64)	
Evers smoking				
No	81.40 (79.85, 82.85)	82.15 (80.35, 83.82)	79.59 (76.51, 82.37)	0.059
Yes	18.60 (15.15, 20.15)	17.85 (16.18, 19.65)	20.41 (17.63, 23.49)	
Physical activity (METs)				
Sufficient	23.60 (22.02, 25.26)	74.84 (72.84, 76.75)	80.44 (77.43, 83.13)	0.007
Insufficient	76.40 (74.74, 77.98)	25.16 (23.25, 27.16)	19.56 (16.87, 22.57)	
Dietary salt intake (gr/day)	9.85 (9.74, 9.95)	9.80 (9.69, 9.91)	9.97 (9.73, 10.21)	0.193
BMI (kg/m^2^)	29.43 (29.22, 29.63)	29.33 (29.09, 29.57)	29.67 (29.29, 30.05)	0.131
WC (cm)	101.07 (100.57, 101.57)	100.38 (99.80, 100.96)	102.87 (101.89, 103.84)	< 0.001
SBP (mmHg)	136.31 (135.54, 137.09)	133.61 (132.78, 134.44)	143.37 (141.72, 145.02)	< 0.001
DBP (mmHg)	81.18 (80.74, 81.61)	80.73 (80.24, 81.21)	82.35 (81.43, 83.27)	< 0.001
FBS (mg/dL)	151.78 (149.42, 154.14)	147.26 (144.68, 149.84)	163.53 (158.43, 168.64)	< 0.001
HbA1c (%)	7.85 (7.78, 7.92)	7.66 (7.58, 7.73)	8.34 (8.19, 8.48)	< 0.001
Glycemic status				
Controlled (HbA1c < 7)	42.91 (40.99, 44.84)	47.48 (45.20, 49.78)	31.04 (27.75, 34.53)	< 0.001
Uncontrolled (HbA1c ≥ 7)	57.09 (55.16, 59.01)	52.52 (50.22, 54.80)	68.96 (65.47, 72.25)	
Total cholesterol (mg/dL)	171.15 (169.51, 172.79)	171.42 (169.54, 173.29)	170.47 (167.14, 173.79)	0.280
LDL‐C (mg/dL)	94.20 (92.81, 95.60)	94.97 (93.36, 96.58)	92.22 (89.46, 94.97)	0.025
HDL‐C (mg/dL)	40.89 (40.50, 41.28)	41.18 (40.72, 41.64)	40.12 (39.39, 40.84)	0.001
TG (mg/dL)	180.70 (176.88, 184.52)	176.41 (171.98, 180.83)	191.90 (184.45, 199.34)	< 0.001
eGFR (mL/min/1.73 m^2^)	86.40 (85.65, 87.15)	91.19 (90.52, 91.85)	73.92 (72.14, 75.70)	< 0.001
Urine albumin/creatinine (mg/g)	57.89 (49.44, 66.34)	8.45 (8.16, 8.74)	187.59 (158.96, 216.21)	< 0.001
Dyslipidemia				
No	9.64 (8.57, 10.82)	10.94 (9.62, 12.43)	6.23 (4.71, 8.20)	< 0.001
Yes	90.36 (89.18, 91.43)	89.06 (87.57, 90.38)	93.77 (91.80, 95.29)	
Hypertension				
No	34.91 (33.11, 36.76)	40.80 (38.61, 43.04)	19.57 (16.85, 22.62)	< 0.001
Yes	65.09 (63.24, 66.89)	59.20 (56.96, 61.39)	80.43 (77.38, 83.15)	
History of CVD				
No	81.86 (80.32, 83.31)	84.57 (82.84, 86.15)	74.81 (71.51, 77.84)	< 0.001
Yes	18.14 (16.69, 19.68)	15.43 (13.85, 17.16)	25.19 (22.16, 28.49)	
Aspirin use				
No	58.74 (56.84, 60.61)	63.87 (61.68, 66.00)	45.36 (41.78, 48.99)	< 0.001
Yes	41.26 (39.39, 43.16)	36.13 (34.00, 38.32)	54.64 (51.01, 58.22)	
Anti‐hypertensive medication				
No hypertensive drug	56.44 (54.53, 58.33)	62.84 (60.64, 64.99)	39.77 (36.28, 43.36)	< 0.001
ACE‐I or ARB	24.13 (22.54, 25.80)	20.98 (19.21, 22.87)	32.34 (29.05, 35.82)	
ACE‐I or ARB + others	9.65 (8.57, 10.86)	7.24 (6.14, 8.52)	15.93 (13.43, 18.80)	
Only other classes	9.78 (8.70, 10.98)	8.94 (7.73, 10.31)	11.96 (9.79, 14.53)	
Statin use				
No	58.19 (56.29, 60.07)	61.63 (59.41, 63.80)	49.21 (45.59, 52.84)	< 0.001
Yes	41.81 (39.93, 43.71)	38.37 (36.20, 40.59)	50.79 (47.16, 54.41)	
Antidiabetic agents use				
No antidiabetic	51.30 (49.39, 53.21)	55.24 (52.99, 57.46)	41.06 (37.57, 44.65)	< 0.001
Only oral	39.70 (37.84, 41.59)	37.63 (35.47, 39.84)	45.08 (41.49, 48.72)	
Only insulin	3.36 (2.73, 4.11)	2.52 (1.67, 3.04)	6.23 (4.72, 8.18)	
Oral + insulin	5.65 (4.82, 6.60)	4.88 (4.00, 5.95)	7.63 (5.89, 9.84)	
Family history of diabetes				
No	47.37 (45.44, 49.31)	47.59 (45.32, 49.87)	46.79 (43.14, 50.48)	0.908
Yes	52.63 (50.69, 54.56)	52.41 (50.13, 54.68)	53.21 (49.52, 56.86)	
TyG index	9.34 (9.32, 9.37)	9.30 (9.27, 9.32)	9.46 (9.41, 9.51)	< 0.001
TyG index (tertiles)				
T1	33.47 (31.68, 35.30)	36.04 (33.90, 38.23)	26.78 (23.68, 30.11)	< 0.001
T2	32.84 (31.08, 34.66)	33.75 (31.66, 35.91)	30.47 (27.26, 33.89)	
T3	33.69 (31.90, 35.53)	30.21 (28.18, 32.32)	42.75 (39.20, 46.38)	
TyG‐BMI	191.17 (189.78, 192.55)	189.72 (188.11, 191.33)	194.93 (192.27, 197.58)	< 0.001
TyG‐BMI (tertiles)				
T1	32.95 (31.17, 34.77)	34.45 (32.34, 36.63)	29.04 (25.87, 32.42)	0.001
T2	33.17 (31.40, 35.00)	33.63 (31.53, 35.78)	32.00 (28.70, 35.49)	
T3	33.88 (32.08, 35.72)	31.92 (29.85, 34.07)	38.96 (35.47, 42.57)	
LAP				
Males	90.63 (87.70, 93.55)	86.69 (83.47, 89.90)	101.11 (94.78, 107.43)	< 0.001
Females	73.47 (70.28, 76.66)	71.04 (67.25, 74.83)	79.64 (73.80, 85.48)	< 0.001
LAP (tertiles)				
Males				
T1	27.74 (25.55, 30.04)	79.30 (75.11, 82.94)	20.70 (17.06, 24.89)	< 0.001
T2	34.37 (32.05, 36.76)	73.31 (69.42, 76.88)	26.69 (23.12, 30.58)	
T3	37.90 (35.52, 40.34)	67.21 (63.29, 70.90)	32.79 (29.10, 36.71)	
Females				
T1	43.63 (40.68, 46.64)	75.46 (71.38, 79.13)	24.54 (20.87, 28.62)	0.050
T2	30.58 (27.87, 33.42)	70.53 (65.30, 75.26)	29.47 (24.72, 34.70)	
T3	25.79 (23.26, 28.49)	66.81 (61.08, 72.09)	33.19 (27.91, 38.92)	
VAI				
Males	4.14 (3.98, 4.30)	3.15 (2.96, 3.33)	3.39 (2.96, 3.33)	< 0.001
Females	3.22 (3.07, 3.36)	3.98 (3.80, 4.15)	4.56 (4.21, 4.92)	< 0.001
VAI (tertiles)				
Males				
T1	27.44 (25.25, 29.74)	29.45 (26.84, 32.20)	22.10 (18.30, 26.44)	0.001
T2	34.82 (32.48, 37.23)	34.87 (32.14, 37.71)	34.67 (30.28, 39.33)	
T3	37.74 (35.37, 40.17)	35.68 (32.95, 38.50)	43.23 (38.58, 48.01)	
Females				
T1	43.82 (40.86, 46.82)	45.51 (41.99, 49.08)	39.52 (34.23, 45.07)	0.128
T2	31.11 (28.39, 33.96)	31.04 (27.84, 34.43)	31.27 (26.30, 36.72)	
T3	25.07 (22.55, 27.78)	23.45 (20.55, 26.61)	29.20 (24.38, 34.54)	
WTI	8.99 (8.97, 9.01)	8.96 (8.93, 8.99)	9.07 (9.03, 9.10)	< 0.001
WTI (tertiles)				
T1	34.06 (32.27, 35.91)	34.81 (32.07, 37.65)	25.55 (21.57, 30.00)	< 0.001
T2	33.12 (31.34, 34.95)	35.05 (32.32, 37.88)	33.24 (28.92, 37.87)	
T3	32.82 (31.05, 34.63)	30.15 (27.56, 32.86)	41.21 (36.59, 45.98)	

Abbreviations: ACE‐I, angiotensin converting enzyme inhibitors; ARB, angiotensin receptor blockers; BMI, body mass index; CVD, cardiovascular disease; DBP, diastolic blood pressure; DKD, diabetic kidney disease; DM, diabetes mellitus; eGFR, estimated glomerular filtration rate; FBS, fasting blood sugar; HbA1c, glycosylated haemoglobin; HDL‐C, high density lipoprotein‐cholesterol; LAP, lipid accumulation product; LDL‐C, low density lipoprotein‐cholesterol; METs, metabolic equivalent of task; SBP, systolic blood pressure; TG, triglyceride; TyG, triglyceride‐glucose; VAI, visceral adiposity index; WC, waist circumference; WTI, waist triglyceride index.

The sex distribution was revealed that 59.26% were male and the majority of the participants (74.01%) resided in urban regions. The DKD proportion was significantly and markedly higher in individuals with < 7 years educational level, being employed, uncontrolled diabetes with dyslipidemia or HTN. In terms of medication use, the DKD proportion was significantly higher among aspirin, ACEI or ARB and only oral antidiabetic users.

The anthropometric indices including TyG, TyG‐BMI, VAI, LAP and WTI were significantly higher in DKD group compared to participants as non‐DKD group, (all *p*‐values < 0.05). Among the three levels of indices, the highest tertile (T3) had the highest proportion of DKD across all five indices, except for LAP and VAI in females.

### 3.2 Univariable Analysis of the Association Between Predictors and DKD

In univariable logistic regression analyses, older age (≥ 60 years), longer diabetes duration (≥ 10 years), higher SBP, higher DBP, presence of hypertension, dyslipidemia, cardiovascular disease, poor glycemic control (HbA1c ≥ 7%), insulin use, anti‐hypertensive drugs, statins and aspirin were all associated with DKD (all *p*‐values < 0.05). On the other hand, participants with 7–11 and ≥ 12 years of education had lower odds of DKD than those with < 7 years of education (both *p*‐values < 0.001) (Table 2). Variables such as dietary salt intake, smoking and family history of diabetes did not show a significant association with DKD (Table 2).

**TABLE 2:** Univariable logistic regression analysis of factors associated with diabetic kidney disease.

Variable	Category	*n* (DKD%)	Model 0	
			OR crude	*p*
Demographic				
Age category (years)	< 40	249 (14.86)	—	—
	40–59	1521 (19.13)	1.36 (0.94, 1.96)	0.106
	≥ 60	1502 (38.95)	3.66 (2.54, 5.26)	< 0.001
Gender	Male	1944 (27.21)	—	—
	Female	1328 (28.92)	1.09 (0.93, 1.27)	0.295
Residency	Rural	882 (29.48)	—	—
	Urban	2390 (27.32)	0.90 (0.76, 1.07)	0.231
Employment status	Unemployed	782 (20.20)	—	—
	Employed	2467 (30.28)	1.72 (1.41, 2.09)	< 0.001
Educational status (years)	< 7	2041 (31.90)	—	—
	7–11	454 (22.69)	0.63 (0.49, 0.80)	< 0.001
	≥ 12	754 (20.03)	0.53 (0.43, 0.66)	< 0.001
Life style				
Ever smoking	No	2671 (27.18)	—	
	Yes	601 (31.11)	1.21 (1.00, 1.47)	0.053
Physical activity (METs)	Sufficient	2494 (29.11)	—	—
	Insufficient	775 (24.13)	0.77 (0.64, 0.94)	0.009
Dietary salt intake (gr/day)	—	—	1.05 (0.97, 1.14)	0.205
DM duration (years)	< 10	2464 (24.03)	—	—
	≥ 10	790 (40.38)	2.14 (1.80, 2.54)	< 0.001
Family history of diabetes	No	1535 (27.75)	—	—
	Yes	1643 (27.94)	1.01 (0.86, 1.18)	0.909
SBP ≥ 140 mmHg (hypertension)	No	1890 (22.22)	—	—
	Yes	1368 (35.75)	1.95 (1.66, 2.28)	< 0.001
DBP ≥ 90 mmHg (hypertension)	No	2486 (26.27)	—	—
	Yes	772 (33.16)	1.39 (1.17, 1.66)	< 0.001
History of CVD	No	2698 (25.57)	—	—
	Yes	568 (38.91)	1.85 (1.54, 2.23)	< 0.001
Medication				
Aspirin use	No	1939 (21.92)	—	—
	Yes	1327 (36.62)	2.06 (1.76, 2.40)	< 0.001
Anti‐hypertensive medications	No hypertensive drug	1850 (20.05)	—	—
	ACE‐I or ARB, *n* (%)	291 (36.70)	2.31 (1.92, 2.78)	< 0.001
	ACE‐I or ARB + others	142 (45.81)	3.37 (2.62, 4.34)	< 0.001
	Only other classes	109 (34.17)	2.07 (1.60, 2.68)	< 0.001
Statin use	No	1923 (23.97)	—	—
	Yes	1344 (33.48)	1.60 (1.37, 1.86)	< 0.001
Antidiabetic medications	No antidiabetic	1702 (23.15)	—	—
	Only oral	1277 (30.85)	1.48 (1.26, 1.75)	< 0.001
	Only insulin	111 (53.15)	3.77 (2.52, 5.62)	< 0.001
	Oral +Insulin	180 (36.67)	1.92 (1.39, 2.66)	< 0.001

*Note:* Robust standard errors were calculated to account for potential heteroscedasticity and clustering effects in the data. Variables shown in grey were excluded from the final multivariable model based on a stepwise selection procedure (*p*‐value = 0.2). Variables retained in the final model represent significant or potentially relevant predictors after survey‐adjusted robust estimation.

Abbreviations: ACE‐I, angiotensin converting enzyme inhibitors; ARB, angiotensin receptor blockers; CVD, cardiovascular disease; DBP, diastolic blood pressure; DKD, diabetic kidney disease; DM, diabetes mellitus; METs, metabolic equivalents of task; OR, odds ratio; SBP, systolic blood pressure.

### 3.3 Correlation Between Quantitative Variables

**FIGURE 2:**
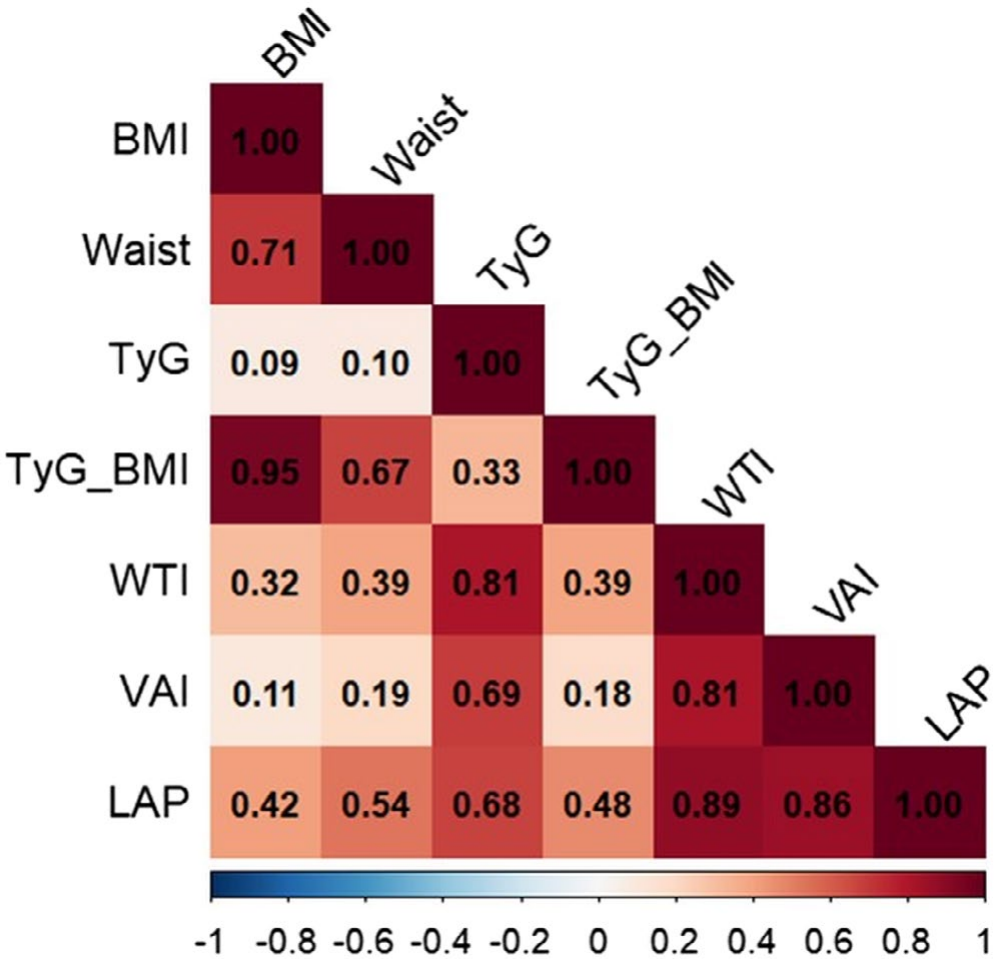
Correlation matrix of metabolic and adiposity‐related indices (BMI, waist circumference, TyG, TyG‐BMI, WTI, VAI and LAP) visualized using a colour gradient to represent the strength of associations. BMI, Body mass index; LAP, Lipid accumulation product; TyG, Triglyceride‐glucose; VAI, Visceral adiposity index; WTI, Waist triglyceride index.

In Figure 2, the correlation matrix shows associations among the studied indices, with correlation coefficients ranging from weak to very strong. TyG index showed weak correlations with primary anthropometric measures, including BMI and waist circumference, whereas moderate to strong correlations were observed between TyG and composite indices related to visceral adiposity, such as WTI, VAI and LAP. In contrast, BMI and waist circumference exhibited strong correlations with each other and with composite indices derived from these anthropometric measures.

### 3.4 Logistic Regression Models of the Associations Between Anthropometric Indices and DKD

In univariable regression analysis, TyG, TyG‐BMI, WTI, VAI and LAP, were associated with higher odds of DKD (all *p*‐values < 0.001). Moreover, after adjusting for the risk factors, by each one‐standard deviation (SD) increase in TyG, TyG‐BMI, WTI, VAI and LAP the odds of DKD increased by 36% (95% CI: 1.24, 1.49), 20% (95% CI: 1.09, 1.31), 27% (95% CI: 1.16, 1.40), 24% (95% CI: 1.14, 1.33) and 27% (95% CI: 1.17, 1.38), respectively (Table 3).

**TABLE 3:** Univariable and multivariable logistic regression models of the associations between anthropometric indices and diabetic kidney disease.

Model	OR crude	*p*	AOR	*p*
Model 1 (TyG)	1.27 (1.18, 1.38)	< 0.001	1.36 (1.24, 1.49)	< 0.001
Model 2 (TyG‐BMI)	1.15 (1.06, 1.24)	< 0.001	1.20 (1.09, 1.31)	< 0.001
Model 3 (WTI)	1.20 (1.11, 1.30)	< 0.001	1.27 (1.16, 1.40)	< 0.001
Model 4 (VAI)	1.15 (1.07, 1.24)	< 0.001	1.24 (1.14, 1.33)	< 0.001
Model 5 (LAP)	1.21 (1.12, 1.30)	< 0.001	1.27 (1.17, 1.38)	< 0.001

*Note:* Robust standard errors were calculated to account for potential heteroscedasticity and clustering effects in the data. The variables TyG, TyG‐BMI, WTI, VAI and LAP were standardized. All models (Models 1–5) were adjusted for Age (years), Gender, Residency, Employment status, Educational status (years), Ever Smoking, Dietary salt intake (gr/day), DM duration (years), SBP, Aspirin use, Anti‐hypertensive medications, Anti‐diabetic medications.

Abbreviations: AOR, Adjusted odds ratio; BMI, Body mass index; LAP, Lipid accumulation product; OR, Odds ratio; TyG, triglyceride‐glucose; VAI, Visceral adiposity index; WTI, Waist triglyceride index.

### 3.5 Discriminative Performance of Anthropometric Indices for Diabetic Kidney Disease

**FIGURE 3:**
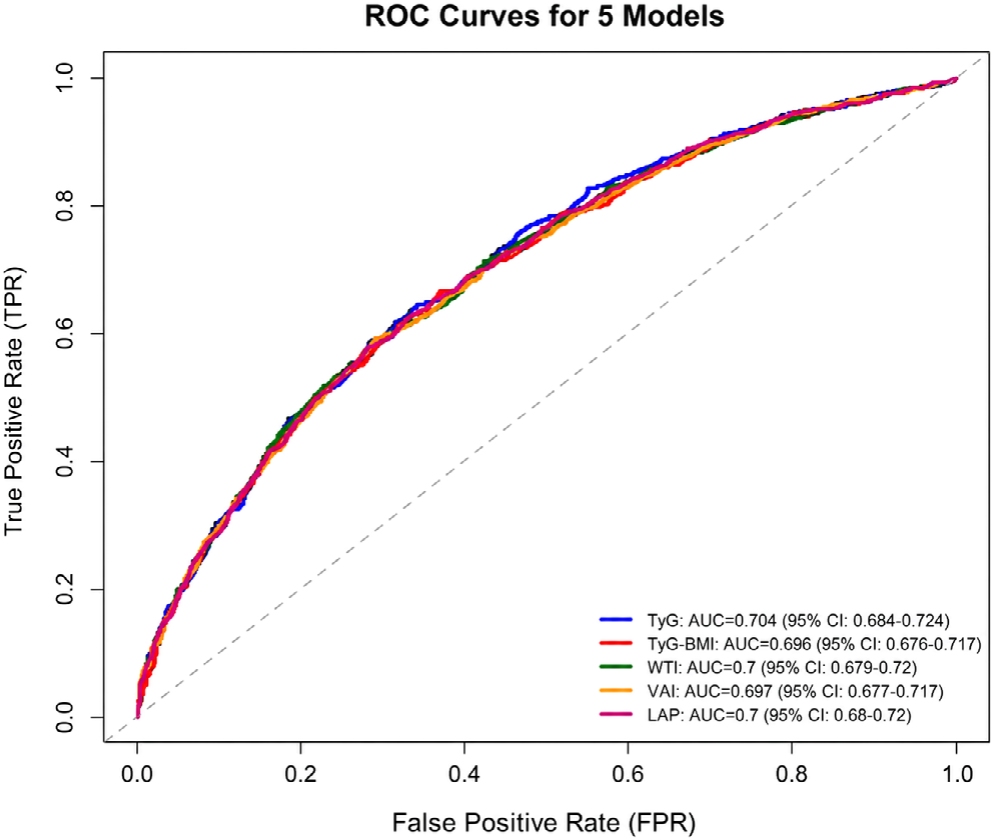
Comparison of predictive power indices for different models.

All five multivariable logistic regression models demonstrated moderate discriminative ability for the presence of DKD, with AUC values ranging from 0.696 to 0.704 (Table 4, Figure 3). The model incorporating the TyG index yielded the AUC (0.704; 95% CI: 0.684–0.724), followed by models including LAP (0.7; 95% CI: 0.679–0.72), WTI (0.7; 95% CI: 0.679–0.72), VAI (0.697; 95% CI: 0.677–0.717) and TyG‐BMI (0.696; 95% CI: 0.676–0.717). The substantial overlap of confidence intervals indicates comparable discriminative performance across models.

**TABLE 4:** Areas under the ROC curve of the anthropometric indices for the detection of diabetic kidney disease.

Model	AUC (95% CI)	Cut‐off (Youden index)	Sensitivity	Specificity	F1 score	Accuracy	Precision
Model 1 (TyG)	0.704 (0.684–0.724)	0.31	0.59	0.71	0.51	0.68	0.44
Model 2 (TyG‐BMI)	0.696 (0.676–0.717)	0.28	0.67	0.63	0.51	0.64	0.41
Model 3 (WTI)	0.7 (0.679–0.72)	0.31	0.59	0.71	0.50	0.68	0.44
Model 4 (VAI)	0.697 (0.677–0.717)	0.32	0.58	0.71	0.51	0.68	0.44
Model 5 (LAP)	0.7 (0.679–0.72)	0.32	0.58	0.72	0.50	0.68	0.44

Abbreviations: AUC, area under the curve; BMI, body mass index; LAP, lipid accumulation product; ROC, receiver operating curve; TyG, triglyceride‐glucose; VAI, visceral adiposity index; WTI, waist triglyceride index.

Pairwise comparisons using DeLong's test for correlated ROC curves showed that the TyG‐based model had a significantly higher AUC compared with VAI (*p* = 0.034), TyG‐BMI (*p* = 0.024) and WTI (*p* = 0.038). No statistically significant differences were observed for the remaining pairwise comparisons. However, after adjustment for multiple testing using the Benjamini–Hochberg false discovery rate (FDR) procedure, none of the pairwise differences remained statistically significant (all adjusted *p* > 0.05), indicating that the initial significant findings were not robust after controlling for type I error inflation. Therefore, overall discriminative performance was considered statistically comparable across all indices (Table 5).

**TABLE 5:** Pairwise comparisons of AUCs between anthropometric indices using DeLong's test with FDR adjustment.

Variable	Comparison	*p*	*p*‐adjusted
LAP	LAP vs. VAI	0.479	0.564
	LAP vs. TyG	0.153	0.306
	LAP vs. TyG‐BMI	0.144	0.305
	LAP vs. WTI	0.508	0.564
VAI	VAI vs. TyG	0.338	0.126
	VAI vs. TyG‐BMI	0.412	0.564
	VAI vs. WTI	0.720	0.720
TYG	TyG vs. TyG‐BMI	0.024	0.126
	TyG vs. WTI	0.038	0.126
TyG‐BMI	TyG‐BMI vs. WTI	0.291	0.484

At the optimal cut‐off values determined by the Youden index, the model including TyG‐BMI demonstrated the highest sensitivity (0.67), whereas the model including LAP showed the highest specificity (0.72). Accuracy was similar across models (range: 0.64–0.68), and F1 scores ranged from 0.50 to 0.51, indicating a moderate balance between sensitivity and precision.

## 4 Discussion

In this large nationwide study of Iranian adults with diabetes, all five selected indices related to insulin resistance and visceral adiposity—TyG, TyG‐BMI, WTI, VAI and LAP—were independently associated with DKD and all showed only moderate discrimination. The findings support the idea that simple, low‐cost metabolic indices may help identify diabetic individuals at higher renal risk, although their predictive performance appears insufficient for clinical decision‐making.

The multifactorial aetiology of DKD is characterized by inter‐related pathophysiological processes, with IR, dyslipidemia, chronic inflammation and oxidative stress representing key contributors [44]. In diabetic individuals, a bidirectional association between IR and kidney damage has been described: IR predicts the progression of kidney dysfunction, while advancing CKD, particularly during uremia state, further aggravates IR [45, 46]. This underscores the clinical importance of assessing IR in patients with diabetes. The TyG index and its derivative TyG‐BMI have been consistently shown to have good discriminatory properties for assessing IR and have been used in various studies to explore the association between IR and DKD [21, 47]. Our findings extend previous evidence by demonstrating that each one‐standard deviation increase in the TyG index was associated with higher odds of DKD (OR = 1.36) after adjustment for multiple confounders, supporting the TyG index as a potential marker of DKD risk.

Although TyG showed the numerically highest AUC, the overlapping confidence intervals and formal ROC comparison indicate that the discriminative performance of the indices was broadly similar. A recent National Health and Nutrition Examination Survey (NHANES 1999–2018)‐based study by Zhang et al. [48] also found that TyG was more consistently associated with diabetic nephropathy than TyG‐BMI and other related indices, supporting the possibility that TyG may capture renal risk more effectively in diabetic populations. Jiang et al. [21] demonstrated that the TyG index was independently associated with DKD among newly diagnosed type 2 diabetes patients, although its diagnostic performance was relatively low (AUC < 0.6). These findings are in line with a recent meta‐analysis of eight studies among Asian populations, which reported a robust association between higher TyG‐index and DKD (OR: 1.68; 95% CI: 1.52–1.86; *p* < 0.001) [49].

TyG‐BMI has been demonstrated to be a more effective marker of IR than TyG index alone, likely because obesity exacerbates IR through mechanisms such as macrophage infiltration, low‐grade inflammation and adipocyte malfunction [50]. In the present study, TyG‐BMI was significantly associated with DKD but exhibited relatively inferior discriminative performance compared with TyG index in distinguishing DKD from non‐DKD individuals. The relatively weaker performance of TyG‐BMI in our study suggests that insulin resistance and central obesity captured by TyG and waist‐related measures may be more closely associated with DKD than general obesity reflected by BMI alone. It has been shown that in the Iranian population with diabetes and metabolic syndrome, central obesity (indicated by WC) and insulin resistance (indicated by TyG index) are significantly stronger, more independent predictors of metabolic complications than general obesity [51]. Because BMI does not differentiate between fat mass and lean mass, its incremental value for risk discrimination may be limited, particularly when BMI distributions were similar across our study groups. Notably, the predictive performance of TyG‐BMI has shown some variability across studies. Consistent with our finding, Sun et al. [14] reported that combining BMI with TyG index resulted in lower discrimination compared with TyG alone, and another study found that although higher TyG‐BMI was associated with DKD risk, its diagnostic value was poor [21].

We found the derivative indices incorporating measures of central obesity—WTI, LAP and VAI—were all independently associated with higher odds of DKD, but their discriminative performance was not strong.

WTI has been proposed as a reliable clinical marker of visceral fat accumulation and lipid abnormalities [22]. Prior research has demonstrated that WTI provides a superior reflection of fat distribution and serves as a simple valuable alternative predictor of IR [52]. In a prospective cohort by Li et al. [53] based on NHANSE database from 2007 to 2018, higher WTI was significantly associated with increased CKD risk and outperformed TyG in predicting CKD. Although Li et al. reported that WTI demonstrated superior discriminatory performance compared with TyG, our findings indicated a slightly higher AUC for TyG. This discrepancy may be explained by several factors. First, the study populations differed; our analysis was limited to Iranian adults with diabetes, whereas the NHANES‐based study likely included a more heterogeneous population with greater metabolic variability. Second, the outcome definitions were not identical because CKD and DKD do not necessarily reflect the same underlying pathophysiological processes. Third, differences in ethnicity, dietary patterns, body fat distribution and cardiometabolic profiles may have influenced the relative performance of these indices. Therefore, the comparative utility of TyG and WTI may depend on the characteristics of the target population and the clinical context in which they are applied. To our knowledge, no studies have specifically explored the correlation between WTI and renal impairment in diabetic patients. In our analysis, each one SD increase in WTI was associated with 1.27 higher odds of DKD after adjusting for potential risk factors, yet its discriminative ability was slightly lower than that of the TyG index. These findings may be attributed to the fact that WTI is partly derived from static anthropometric measurements and may not capture the full spectrum of dynamic metabolic disturbance that is more directly reflected by the TyG index, which integrates both glycemic and lipid components.

In our studied population, the observed effect sizes of VAI and LAP—corresponding to 24% and 27% higher odds of DKD per each SD increase, respectively‐ indicate clinically meaningful independent association between these indices and DKD. Although, LAP achieved slightly higher AUC than VAI (0.700 vs. 0.697), both indices exhibited moderate discriminative capacity. Several prior cohort and cross‐sectional studies have shown that VAI and LAP have independent positive association with DKD, consistent with our results [26, 27, 53]. A recent meta‐analysis reported that VAI and LAP are significant predictors of DKD, however they exhibited limited ability to differentiate patients with DKD from individuals without‐DKD in clinical practice (pooling AUCs < 0.7) [54]. This association between VAI or LAP and DKD could be explained by the central role of visceral obesity in metabolic dysregulation [55].

Visceral adipocytes are capable of secreting large amounts of free fatty acids, which reach the liver and impair insulin signalling through several pathways, thereby contributing to the development of IR [56], a recognized risk factor for DKD development and progression [57]. In addition, in the state of central obesity, visceral adipocytes can secrete excessive various pro‐inflammatory cytokines, including tumour necrosis factor‐α (TNF‐α) and interleukin‐6 (IL‐6), leading to chronic low‐grade inflammation and exerting direct harmful effects on renal tissue [58]. Although lipids are essential for cell function, non‐adipose tissue cells including renal cells have limited capacity to handle lipid overload and, therefore, are vulnerable to lipotoxicity [59]. Moreover, the literature supports that high salt intake can impair visceral adipose tissue homeostasis and that salt‐related visceral adipose dysfunction may contribute to systemic and renal injury. Therefore, high sodium intake may synergize with visceral adiposity to accelerate DKD progression through combined effects on glomerular hyperfiltration, inflammation and adipose tissue dysfunction [60].

Indices such as VAI and LAP which integrates both anthropometric measurements (WC and BMI) and blood biochemical indexes such as serum TG levels and HDL‐C, mirror the combined burden of visceral adiposity and dyslipidemia and exhibits consistent association with DKD in different populations [27]. Their simplicity, low cost and feasibility in routine care and large‐scale surveys make them attractive tools for risk stratification, even though their stand‐alone discriminative performance is only moderate.

This study has several strengths, including a large nationwide representative sample of Iranian adults with diabetes, the simultaneous evaluation of five selected anthropometric indices, and the use of survey‐weighted multivariable analyses with adjustment for a broad range of demographic, lifestyle, clinical and medication‐related factors. However, several limitations should be considered. The cross‐sectional design precludes any causal inference or assessment of temporality between the indices and DKD. We were unable to adjust for total caloric intake (a major determinant of circulating triglycerides and waist circumference), detailed macronutrient composition, and specific antidiabetic drug classes such as sodium‐glucose‐transport 2 (SGLT2) inhibitors and glucagon‐like peptide‐1 (GLP‐1) receptor agonists, which may have influenced the observed associations. Furthermore, diabetes duration was modelled categorically, which may have reduced precision. However, the moderate discriminatory performance of the indices suggests they should be considered adjunctive rather than standalone predictors of DKD.

## 5 Conclusion

In conclusion, TyG, TyG‐BMI, WTI, VAI and LAP were all associated with DKD in Iranian adults with diabetes, but their ability to discriminate DKD was only moderate. These indices may be useful as low‐cost adjunct markers for identifying high‐risk individuals, particularly in resource‐limited settings, but they should be interpreted alongside established clinical risk.

## Author Contributions

**Mahnaz Pejman Sani:** conceptualization, project administration, supervision, writing – review and editing. **Mehrsadat Miri:** writing – original draft. **Ali Golestani:** data curation, writing – review and editing, validation. **Shahrzad Mohseni:** investigation, writing – review and editing. **Samaneh Akbarpour:** methodology, validation, writing – review and editing. **Ozra Tabatabaei‐Malazy:** conceptualization, project administration, writing – review and editing. **Maryam Karimi Ghahfarokhi:** formal analysis, writing – review and editing, data curation.

## Funding

The authors have nothing to report.

## Ethics Statement

The present study was reviewed and approved by Research Ethics Committees of Endocrine and Metabolism Research Institute at Tehran University of Medical Sciences, Tehran, Iran (ID: IR.TUMS.EMRI.REC.1403.092). This study was conducted in accordance with relevant institutional guidelines, regulations and the Declaration of Helsinki.

## Consent

Written informed consent was obtained from all enrolled participants.

## Conflicts of Interest

The authors declare no conflicts of interest.

## Data Availability

The data that support the findings of this study are available from the corresponding author upon reasonable request.
